# Faunal and Ecological Analysis of Gamasid Mites (Acari: Mesostigmata) Associated with Small Mammals in Yunnan Province, Southwest China

**DOI:** 10.3390/insects16030305

**Published:** 2025-03-15

**Authors:** Peng-Wu Yin, Pei-Ying Peng, Xian-Guo Guo, Wen-Yu Song, Tian-Guang Ren, Ya-Fei Zhao, Wen-Ge Dong, Dao-Chao Jin

**Affiliations:** 1 Institute of Pathogens and Vectors, Yunnan Provincial Key Laboratory for Zoonosis Control and Prevention, Dali University, Dali 671000, China; 2 Institute of Microbiology, Qujing Medical College, Qujing 655100, China; 3 School of Government Administration, Baoshan University, Baoshan 678000, China; 4 Institute of Entomology, Guizhou University, Guiyang 550025, China

**Keywords:** Acari, ectoparasite, faunal distribution, gamasid mite, species diversity, Yunnan of China

## Abstract

Gamasid mites (Acari: Mesostigmata) are ecologically diverse arthropods, many of which act as vectors for zoonotic diseases such as rickettsial pox and hemorrhagic fever with renal syndrome. This study investigates the faunal and ecological patterns of gamasid mites across five zoogeographic microregions in Yunnan Province, China, a biodiversity hotspot with complex topography. From 1990 to 2022, 18,063 small mammal hosts (primarily rodents) were surveyed, yielding 167 mite species (141,501 specimens). The key findings include the following: (1) Low host specificity: most mite species parasitized >10 host species, with *Laelaps nuttalli*, *L. echidninus*, *Dipolaelaps anourosorecis*, *L. guizhouensis*, *L. turkestanicus,* and *L. chini* dominating (>76.59% abundance). (2) Environmental heterogeneity: mountainous and outdoor habitats exhibited higher mite diversity than flatland/indoor environments. (3) Zoonotic risks: thirteen vector species with low host specificity were identified, potentially amplifying disease transmission. (4) Ecological niche dynamics: high niche overlaps (e.g., *Laelaps guizhouensis* vs. *L. xingyiensis*: *O_ik_* = 0.997) and positive interspecific correlations (e.g., *L. echidninus* vs. *L. nuttalli*: *R* = 0.97, *p* < 0.01) suggest co-occurrence trends on shared hosts. (5) Biogeographic patterns: mite communities were clustered distinctly by microregion, with the highest similarity being obtained between western/southern plateaus (IV and V) and unique diversity in the Hengduan Mountains (I). (6) Chao 1 estimation predicted 203 total mite species in Yunnan, 36 of which were undetected in the current sampling. These results highlight the interplay of biogeography, host ecology, and environmental factors in shaping mite distributions, with implications for zoonotic disease surveillance in biodiverse regions.

## Simple Summary

Gamasid mites are a large group of arthropods, and some of them can serve as vectors or potential vectors of some zoonotic diseases. Yunnan Province of southwest China is an important focus of many zoonotic diseases, with a complicated topography and high biodiversity. The field investigations conducted in 40 survey sites of Yunnan covered five zoogeographical microregions (I, II, III, IV, V). The mites collected from rodents and other sympatric small mammals (hosts) were identified to species level under a microscope, and a series of statistical methods and visualization models were used. Collected from 18,063 hosts, 141,501 mites were identified as 167 species, showing a high level of species diversity. Most mite species showed low host specificity, being found on more than 10 host species. The mite community diversity in the mountainous landscape and outdoor habitat was obviously higher than that in the flatland landscape and indoor habitat, with obvious environmental heterogeneity. The coexistence of 13 vector mite species with low host specificity may increase the potential risk of zoonoses’ transmission. Some mite species showed high similarity in host selection with high niche overlaps. The mite faunae in microregions IV and V showed the highest similarity, which is associated with the specific locality, topography, and climate types in different microregions. Some mite species showed a tendency to choose the same hosts, with a positive correlation. The expected total number of mite species in Yunnan was estimated to be 203 species, 36 species more than actually identified 167 species.

## 1. Introduction

Gamasid mites are a large group of arthropods with abundant species and different ecological behaviors. The taxonomic status of gamasid remains controversial, and different scholars have proposed different taxonomic systems. Based on the taxonomic system established by Krantz in 1978 [1], which has been accepted by many scholars, gamasid mites belong to the suborder Mesostigmata (or Gamasida), and subclass Acari in the class Arachnida or Arthropoda [1]. The ecological behaviors of gamasid mites vary from species to species. Some gamasid mites are free-living, while others are parasitic, including endoparasitic and ectoparasitic mites. Ectoparasitic gamasid mites are the most common parasitic variety, and they have an extensive range of hosts, including mammals, birds, reptiles, amphibians, and even other arthropods. Rodents (rats, mice, voles, etc.) and other sympatric small mammals (e.g., shrews and tree shrews) are the most common and important hosts of ectoparasitic gamasid mites [2,3,4]. The ectoparasitic gamasid mites found on rodents and other small mammals are of medical significance and an important part of medical arthropods. Besides directly invading and biting humans to cause dermatitis, some species of ectoparasitic gamasid mites can serve as a vector or potential vector of some zoonotic diseases (zoonoses), such as rickettsial pox, hemorrhagic fever with renal syndrome (HFRS) and some other zoonotic diseases [5,6,7,8]. Rodents and other sympatric small mammals are not only the most important hosts of ectoparasitic gamasid mites, but also the most important infectious source of many zoonotic diseases, such as plague, murine typhus, leptospirosis, scrub typhus, and HFRS. Through the biting activity, the pathogens of rickettsial pox, HFRS and some other zoonotic diseases can be transmitted from rodents and other small mammals to humans [9]. In field surveys, the gamasid mites collected from the body surface of small mammals were not all ectoparasitic species. Some free-living gamasid mites can be occasionally collected after “losing their way” and climbing onto the body surface of small mammals. In the present study, bats (the order Chiroptera) were not included because the ecological behaviors and collection methods of bats (the order Chiroptera) are obviously different from those of rodents.

Yunnan Province (21°08′–29°15′ N, 97°31′–106°11′ E) is located in the southwest of China, and is a mountainous region with a vast territory (394,100 km^2^), complicated topographic landform, and high biodiversity. The altitude of Yunnan ranges from the lowest altitude, found in Hekou (76.4 m), to the highest, at the summit of Meili Snow Mountain (6740 m). Being rich in biological resources, Yunnan is known as the “Kingdom of Plants and Animals” in China. The species of plants and animals found in Yunnan account for more than 50% of the total species in the whole of China [10,11,12]. Western Yunnan belongs to the Hengduan Mountainous Region and its extension area, and its topography, geomorphology, and ecological environment are extremely complex, with high biodiversity. In particular, the Three Parallel Rivers Area (26–29° N, 98–100° E) in the northwest of Yunnan is a World Nature Heritage Site. In this area, there are three rivers (the Jinshajiang, Lancangjiang, and Nujiang rivers) originating from the Tibetan Plateau, flowing from the north towards the south. The rivers pass in parallel through four main massive mountains (the Gaoligong, Nushan, Yunling, and Shaluli Mountains) without intersection through a series of deep valleys, forming a very complex longitudinal mountain–valley landscape. The Three Parallel Rivers Area is a hotspot of biodiversity in Asia, with high species diversity [13,14]. The geographical location, unique landscape, complex topography, and diversified ecological environment of Yunnan provide a good place to study the species diversity and fauna of animals, including ectoparasitic gamasid mites and their small mammal hosts.

Fauna refers to specific groups of animals that have developed over a certain historical period due to geographic isolation, and faunal studies aim to reveal the species composition and distribution patterns of an animal group within a specific geographic region [15,16,17]. A series of field surveys and the taxonomic identification of a large number of species must be carried out before the faunal analysis, and taxonomic techniques are particularly important in faunal studies [18,19]. Although there were some literature reports related to the fauna of gamasid mites, many previous studies had their own limitations, and the number of gamasid species documented was very limited. Some faunal reports were just based on the historical literature, without formal and systematic investigations, e.g., the faunal reports of gamasid mites from provincial regions of Ningxia, Shaanxi, Qinghai, and Hubei in China [20,21,22,23,24,25]. Some of the faunal literature was based on investigations within a narrow geographical area, e.g., the faunal investigations of gamasid mites at the Gaoligong and Dandanglika Mountains located in the northwest of Yunnan Province [26]. Some others came from sporadic surveys, which lacked formal and scientific sampling [27,28,29,30,31,32,33,34]. Based on a series of field investigations and the taxonomic identification of abundant specimens in 40 survey sites of Yunnan Province between 1990 and 2022, the present study systematically reports the faunal distribution of gamasid mites associated with rodents and other sympatric small mammals in Yunnan Province of southwest China for the first time.

## 2. Materials and Methods

### 2.1. Study Areas

**Figure 1:**
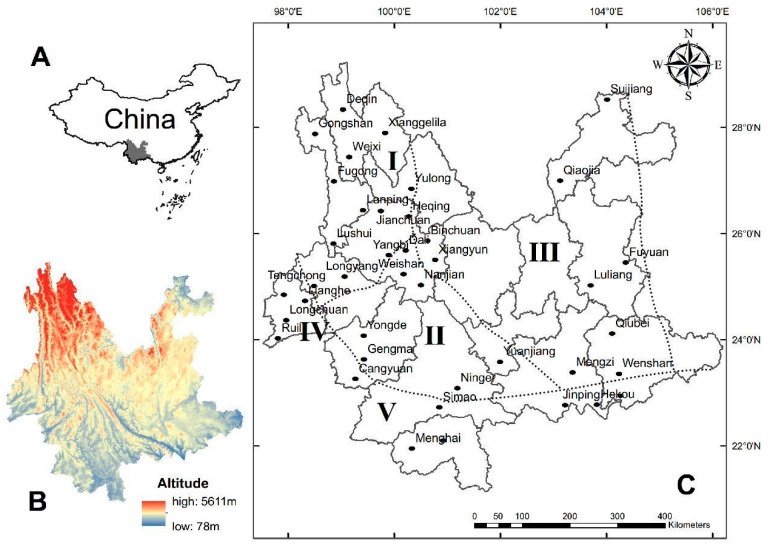
The five zoogeographical microregions (I, II, III, IV, V) and 40 survey sites (counties) of Yunnan Province in southwest China (1990–2022). (**A**) Location of Yunnan Province on the map of China; (**B**) topographic map of Yunnan Province; (**C**) the 40 survey sites (counties) in the five zoogeographical microregions of Yunnan Province.

According to zoogeographical divisions, Yunnan Province is located in the Sino–Indian Subrealm under the Oriental Realm. Within the subrealm, most parts of Yunnan belong to the Southwest Mountain Subregion in the Southwest Region of China, and a small part in the south belongs to the Southern Yunnan Mountain Subregion in the South Region of China. Within the two subregions (Southwest Mountain Subregion and Southern Yunnan Mountain Subregion), there are five zoogeographical microregions in Yunnan, namely the Middle Microregion of the Hengduan Mountains (I), the Southern Microregion of the Hengduan Mountains (II), the Eastern Plateau Microregion of Yunnan (III), the Western Plateau Microregion of Yunnan (IV), and the Southern Mountainous Microregion of Yunnan (V). The first three microregions (I, II, and III) belong to the Southwest Region and the latter two microregions (IV and V) belong to the South Region (Table 1, Figure 1) [35,36]. According to the territory, topography, geomorphology, altitude gradients, and other characteristics of the five zoogeographic microregions, a series of stratified sampling investigations were successively conducted in 40 counties (survey sites) of Yunnan Province between 1990 and 2022. The number of survey sites was inconsistent in the five microregions. There were 13 survey sites (the most) in the Middle Microregion of Hengduan Mountains (I) due to the extremely complicated topography, geomorphology, and altitude gradients of this microregion, where the Three Parallel Rivers Area, a World Nature Heritage Site, is located. In contrast, there were only four survey sites (the fewest) in the Western Plateau Microregion of Yunnan (IV) due to its having the narrowest territory (Figure 1).

### 2.2. Collection and Identification of Gamasid Mites and Their Hosts

Rodents and other sympatric small mammals were mainly trapped with mousetraps (18 × 12 × 9 cm, Guixi Mousetrap Apparatus Factory, Guixi, Jiangxi, China). Mousetraps were set in dwelling areas (human house, poultry house, pig pen, cattle pen, stable, etc.), farmland, scrubland, and woodland in the afternoon or evening, and then checked the following morning. Each trapped small mammal host was placed in an individual pre-marked cloth bag and then transferred to the field temporary laboratory, where the host was anesthetized with ether [2,36]. With the help of a magnifier, gamasid mites were collected from the body surface of each host over a large white tray, and the collected mites were preserved in labeled vials containing 70% or 75% ethanol. After the collection of gamasid mites, each host (small mammal) was conventionally identified to species level according to its morphology [2,37]. In the laboratory, all the gamasid mites were separately mounted onto glass slides with Hoyer’s medium. After the dehydration and transparency process, all the mounted mite specimens were finally identified to species level under a microscope [2,18].

The taxonomic identification of gamasid mites in this study was primarily based on the following references: *A Manual of Acarology* (Third Edition); *Economic Insect Fauna of China. Fasc 17*, *Acari: Gamasina*; *Economic Insect Fauna of China. Fasc. 40*, *Acari: Dermanyssoidese*; and *Soil Gamasida from Northeast China*. Additional taxonomic literature on gamasid mite identification, including descriptions of new species and new records, was also consulted [18,19,38,39]. Throughout the entire study period (1990–2022), our research team maintained identical sampling and analytical methods, which included the following: standardized rodent trapping protocols; consistent specimen preparation and identification procedures; uniform statistical analyses across all datasets.

The use of animals (including animal euthanasia) for our research was officially approved by the Animals’ Ethics Committee of Dali University, approval code: DLYXY1990-0109, DLXY2001-1116 and DLDXLL2020-1104, approval date: 9 January 1990, 16 November 2001 and 4 November 2020. Voucher mites and representative mammals are deposited in the specimen repository of the Institute of Pathogens and Vectors, Dali University, China.

### 2.3. Statistics on Constituent Ratios and Community Indexes

The constituent ratio (*C_r_*) was used to calculate the percentage of a certain gamasid mite species on small mammal hosts, and the prevalence (*PM*) was used to calculate the percentage of infested hosts [40,41,42]. In addition to the number of species, three community diversity indexes were introduced to compare the faunal differences in gamasid mites and their small mammal hosts in the five zoogeographical microregions of Yunnan Province. These community diversity indexes were the Margalef richness index (*R*), Simpson diversity index (*D*), and Shannon–Wiener diversity index (*H*) [43,44,45].

$$C_{r}=\frac{N_{i}}{N}\times 100\%;\;PM=\frac{H_{i}}{H}\times 100\%;\;R=\frac{S-1}{\ln N};\;D={\sum_{i=1}^{S}\left(\frac{N_{i}}{N}\right)}^{2};\;H=-\sum_{i=1}^{S}\frac{N_{i}}{N}\ln\frac{N_{i}}{N}$$

In the above formulas, *N_i_* is the number of a certain gamasid mite species (species *i*), *N* is the total number of all gamasid mites, and *S* stands for the richness of gamasid mite species, which is actually the number of species in the gamasid mite community. *H_i_* is the number of small mammal hosts infested with gamasid mites; *H* is the total number of small mammal hosts.

### 2.4. Analysis of the Relationship Between Gamasid Mites and Their Hosts

A bipartite network analysis in the bipartite R package was used to visualize the relationship between the main species of gamasid mites and their small mammal hosts.

### 2.5. Analyses of the Ecological Niches of Gamasid Mites

Levins-based ecological niche width (*B_i_*) was used to analyze the niche widths of gamasid mite species of different resource series, host species, or environmental gradients [46,47]. Pianka-based overlap index (*O_ik_*) was used to measure the niche overlaps among different gamasid mite species [48,49,50].

$$B_{i}=\frac{1}{S\sum_{n=1}^{S}p_{in}^{2}}\times 100;\;O_{\mathrm{ik}}=\frac{\sum_{j=1}^{n}P_{\mathrm{ij}}P_{\mathrm{kj}}}{\sqrt{\sum_{j=1}^{n}P_{ij}\sum_{j=1}^{n}{P_{kj}}^{2}}}$$

In the above formulas, *B_i_* = the niche width of gamasid mite species *i*; *P_in_* = the proportion of gamasid mite species *i* in resource series *n* (host species *n* or environmental gradient *n*); *S* = the number of total resource series; *O_ik_* = the niche overlap between gamasid mite species *i* and *k*; *P_ij_* = the proportion of gamasid mite species *i* in resource series *j*; and *P_kj_* = the proportion of gamasid mite species *k* in resource series *j*. The value range of *O_ik_* is: 0 < *O_ik_* < 1. “*O_ik_* = 1” means a complete niche overlap, and “*O_ik_* = 0” means no niche overlap at all.

### 2.6. Analyses of the Interspecific Relationships of Gamasid Mites

The correlation coefficient (*R*) was used to measure the interspecific relationship between any two gamasid mite species in the selection of hosts [51,52].

$$R=\frac{\sum_{j=1}^{N}\left(x_{\mathrm{ij}}-{\bar{x}}_{i})(x_{\mathrm{kj}}-{\bar{x}}_{k}\right)}{\sqrt{\sum_{j=1}^{N}(x_{\mathrm{ij}}-{\bar{x}}_{i}{)}^{2}\sum_{j=1}^{N}(x_{\mathrm{kj}}-{\bar{x}}_{k}{)}^{2}}}$$

In the above equation, *R* = the correlation coefficient between species *i* and *k* of gamasid mites; *N* = the number of samples; *x_ij_* and *x_kj_* represent the number of gamasid mite species *i* and *k* in sample *j*; and ${\bar{x}}_{i}$ and ${\bar{x}}_{k}$ represent the average number of gamasid mite species *i* and *k* across all the samples. The value of *R* ranges from −1 to 1 [−1, 1], representing the negative and positive correlations. The statistical significance of *R* is determined by *t*-test (degrees of freedom = *N* − 2).

### 2.7. Faunal Similarity Analysis

The hierarchical cluster analysis was used to analyze the faunal similarity of gamasid mites and their small mammal hosts in the five zoogeographic microregions of Yunnan Province. Before the faunal similarity calculation, an original data matrix was established, which included the following variables: the average numbers of species ($\bar{S}$) of small mammal hosts and gamasid mites, overall infestation indexes (*PM*, *MA* and *MI*) of all gamasid mites on all hosts, constituent ratios (*C_r_*) of dominant species of hosts and mites, and average community indexes ($\bar{D}$, $\bar{H}$, $\bar{R}$) of hosts and mites in different zoogeographic microregions. Based on Z-score standardization, the squared Euclidean distance (*d_ij_*) was used to calculate the faunal similarity of gamasid mites among the five different zoogeographic microregions. The clustering result was presented and visualized using a dendrogram through the centroid clustering methods. All the calculations and visualizations were performed using the Origin software 2024a (a statistical and mapping software). The formula for the squared Euclidean distance (*d_ij_*) is as follows:

$$d_{\mathrm{ij}}=\sum_{k=1}^{m}{\left|x_{ki}-x_{kj}\right|}^{2}$$

In the above equation, *d_ij_* = the similarity of gamasid mite faunae between any two of the five different zoogeographic microregions, fauna *i* and *j*; *x_ki_* = the *k*th variable in fauna *i*; and *x_kj_* = the *k*th variable in fauna *j*.

### 2.8. Estimation of Total Species

Chao 1 method, which is based on rare species, was used to roughly estimate the total number of gamasid mite species in Yunnan Province, and the calculation formula is as follows:

$$S^{*}=S_{obs}+\frac{a^{2}}{2b}$$

In the above formula, S^*^ = the expected number of species; *S_obs_* = the actual number of species; *a* = the number of species with only one individual; and *b* = the number of species with only two individuals [53,54].

## 3. Results

### 3.1. Species Diversity and Faunal Distribution of Small Mammal Hosts

**Figure 2:**
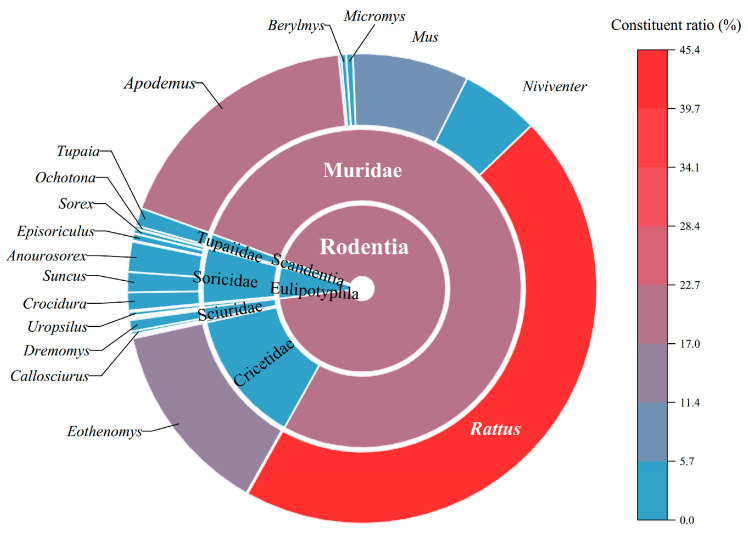
A rising sun map for the constituent ratios (*C_r_*) of small mammal hosts (individuals) at levels of order, family, and genus in Yunnan Province, southwest China (1990–2022).

A series of field investigations were carried out in 40 survey sites (counties) of Yunnan Province between 1990 and 2022. The main original data came from systematic field surveys between 2001 and 2022, and only a small portion of the data were from sporadic surveys prior to 2001 (1990–2000). A total of 18,063 small mammal hosts captured were identified as 78 species, 38 genera, and 11 families in five orders: Rodentia, Eulipotyphla, Lagomorpha, Scandentia, and Carnivora (Table 2). At the order level, the order Rodentia (rodents) formed the majority of the hosts in Yunnan, accounting for 92.53% (16,714/18,063) of all the small mammal hosts. The family Muridae and the genus *Rattus* under the order Rodentia comprised the majority of all the hosts at the family and genus levels (Figure 2). Among the 78 host species identified, the total constituent ratios (*C_r_*) of three dominant host species reached 62.17% (11,230/18,063). These dominant host species were all rodents: the rat *Rattus tanezumi* (*C_r_* = 29.82%, 5387/18,063), the mouse *Apodemus chevrieri* (*C_r_* = 12.27%, 2216/18,063), the vole *Eothenomys miletus* (*C_r_* = 12.04%, 2175/18,063), and the rat *R. norvegicus* (*C_r_* = 8.04%, 1452/18,063).

**Figure 3:**
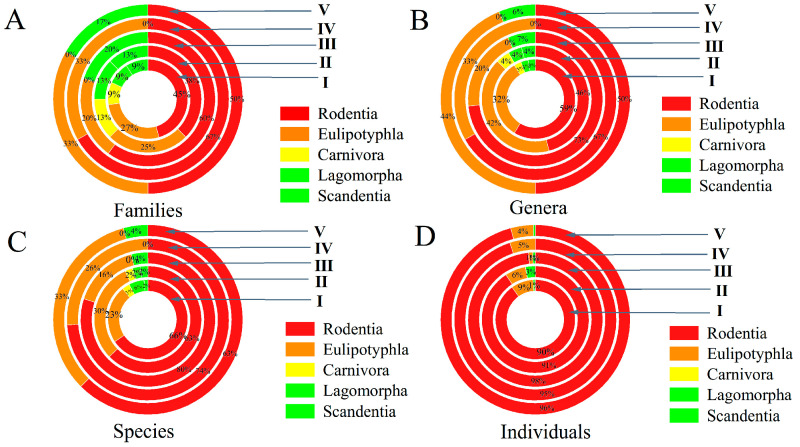
Rising sun maps for the constituent ratios (*C_r_*) of small animal hosts (numbers of families, genera, species or individuals) in the five zoogeographical microregions of Yunnan Province, southwest China (1990–2022). (**A**) The constituent ratios (*C_r_*) of family numbers of hosts; (**B**) the constituent ratios (*C_r_*) of genus numbers of hosts; (**C**) the constituent ratios (*C_r_*) of species numbers of hosts; (**D**) The constituent ratios (*C_r_*) of host individuals: I = Middle Microregion of the Hengduan Mountains; II = Southern Microregion of the Hengduan Mountains; III = Eastern Plateau Microregion of Yunnan; IV = Western Plateau Microregion of Yunnan; V = Southern Mountainous Microregion of Yunnan.

In the five zoogeographical microregions of Yunnan, the numbers of families, genera, and species of small mammal hosts in the order Rodentia were higher than those in other orders. The number of individuals belonging to the order Rodentia was notably higher than other orders (Figure 3). In order to make a comprehensive comparison of species diversity in different geographical microregions, four community indexes were introduced in the present study. Due to the inconsistent numbers of survey sites and small mammal hosts (host samples) in the five zoogeographical microregions, 500 individuals of small mammal hosts were grouped to calculate the average values of the community indexes in each microregion. The hosts with ≤250 <500 individuals constituted an independent last group. The hosts with <250 individuals merged into the former group. The calculated community indexes include the average number of species ($\bar{S}$), average Margalef richness index ($\bar{R}$), average Simpson diversity index ($\bar{D}$), and average Shannon–Wiener diversity index ($\bar{H}$). The results showed that the average values of $\bar{S}$ (15.09), $\bar{R}$ (2.26), and $\bar{H}$ (1.68) were highest in microregion I (Table 3). The dominant host species were also different in the five microregions. *Apodemus chevrieri* was the most dominant host species in microregion I, with *C_r_* = 21.77%, and *R. tanezumi* was the exclusive dominant host species in microregion V, with *C_r_* = 75.24% (Table 4).

### 3.2. Species Diversity and Faunal Distribution of Gamasid Mites

**Figure 4:**
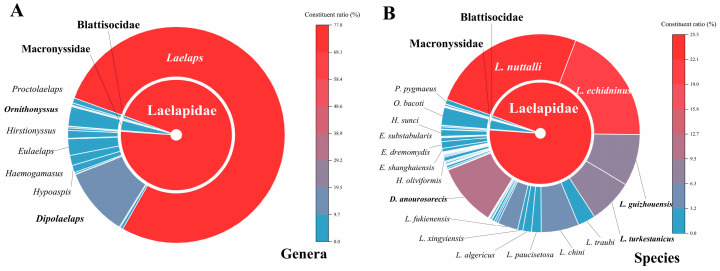
Rising sun maps for the constituent ratios (*C_r_*) of gamasid mites (individuals) at the levels of family, genus, and species in Yunnan Province, southwest China (1990–2022). (**A**) The constituent ratios (*C_r_*) of gamasid mites (individuals) at the levels of family and genus; (**B**) the constituent ratios (*C_r_*) of gamasid mites (individuals) at the levels of family and species.

A total of 143,698 gamasid mites were collected from the body surfaces of 18,063 small mammal hosts, of which 141,501 were identified as belonging to 167 species and 41 genera in 14 families. The rest of the 2197 mites remained unidentified due to unclear morphological structures in some specimens and some suspected new species. The 14 families identified were Laelapidae, Dermanyssidae, Macronyssidae, Aceosejidae, Ameroseiidae, Parasitidae, Phytoseiidae, Ascidae, Parholaspidae, Macrochelidae, Pachylaelaptidae, Rhodacaridae, Eviphididae, and Blattisocidae. Laelapidae exhibited the highest abundance in terms of species and individuals, with 17 genera, 107 species, and 135,110 individuals, and Macronyssidae came next, with 2 genera, 2 species, and 4135 individuals. Phytoseiidae had the lowest number of species and individuals, with only one genus, one species, and one individual (Table 5, Figure 4).

**Figure A1:**
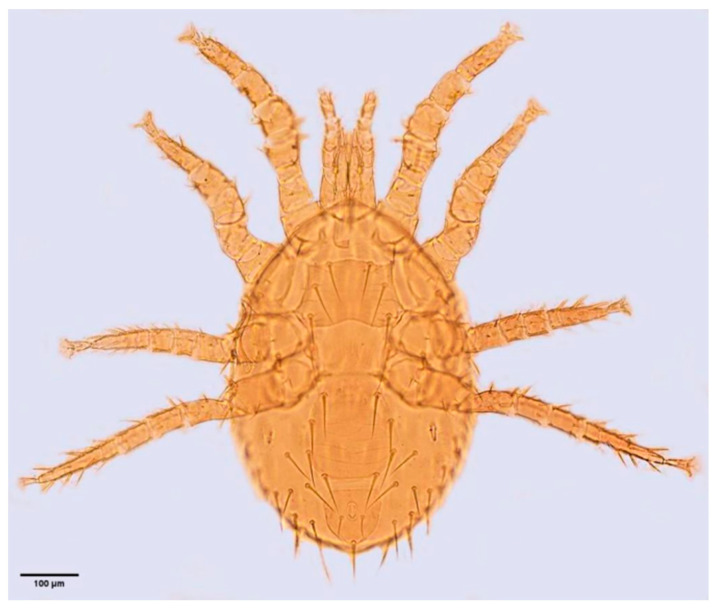
*Laelaps nuttali* photograph; female mite; center view (scale = 100 μm).

**Figure A2:**
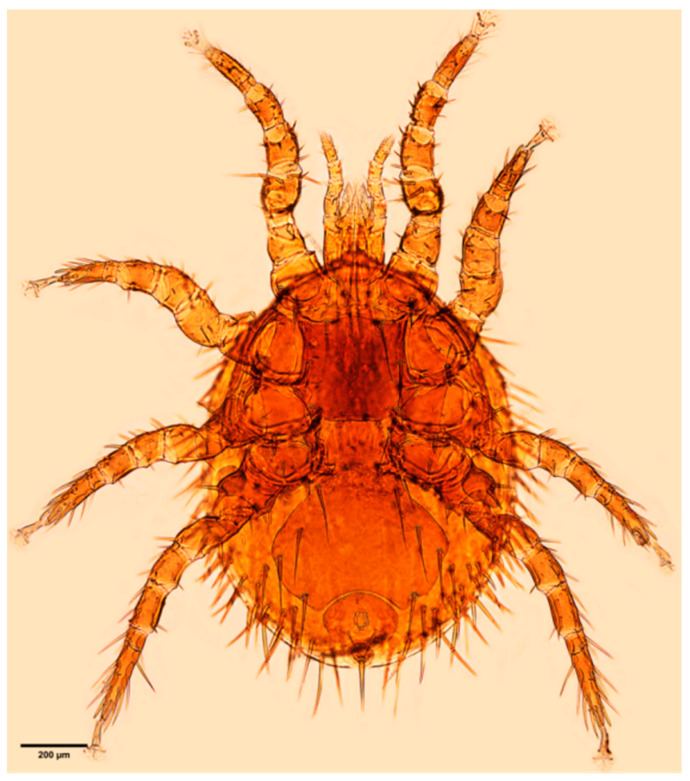
*Laelaps echidninus* photograph; female mite; center view (scale = 200 μm).

**Figure A3:**
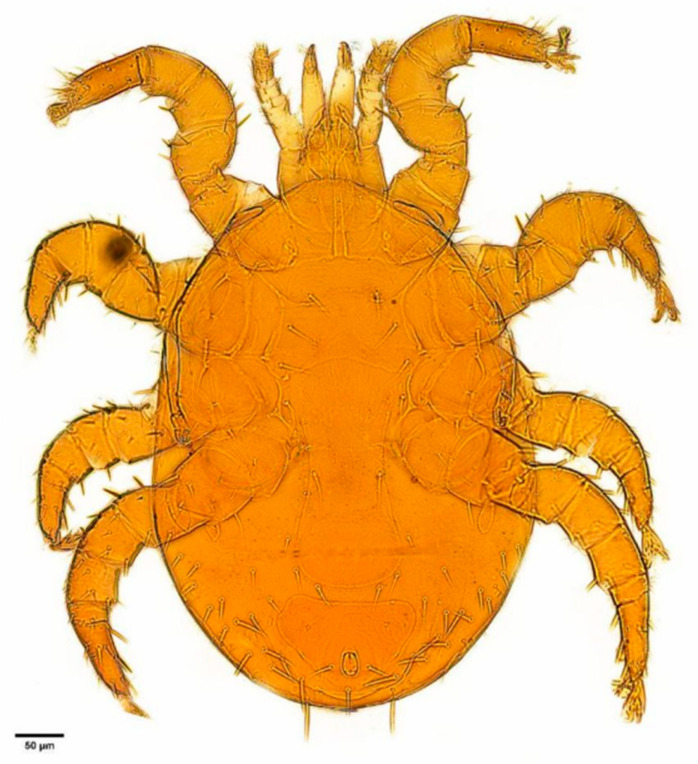
*Dipolaelaps anourosorecis* photograph; female mite; center view (scale = 50 μm).

**Figure A4:**
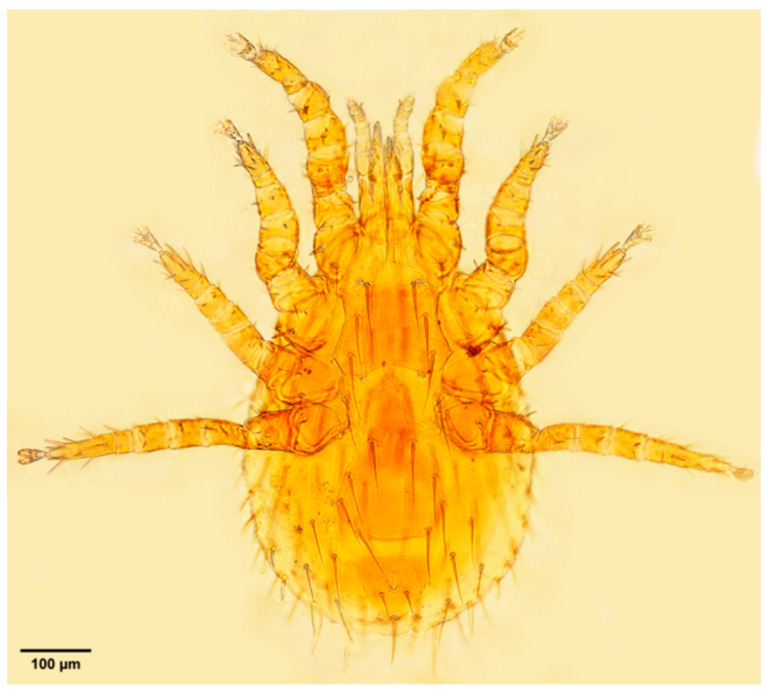
*Laelaps guizhouensis* photograph; female mite; center view (scale = 100 μm).

**Figure A5:**
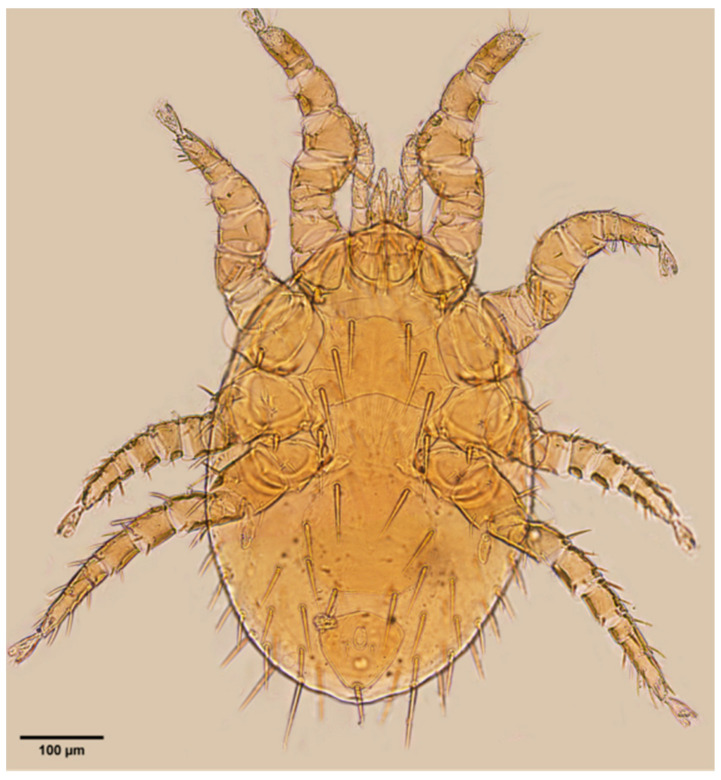
*Laelaps turkestanicus* photograph; female mite; center view (scale = 100 μm).

**Figure A6:**
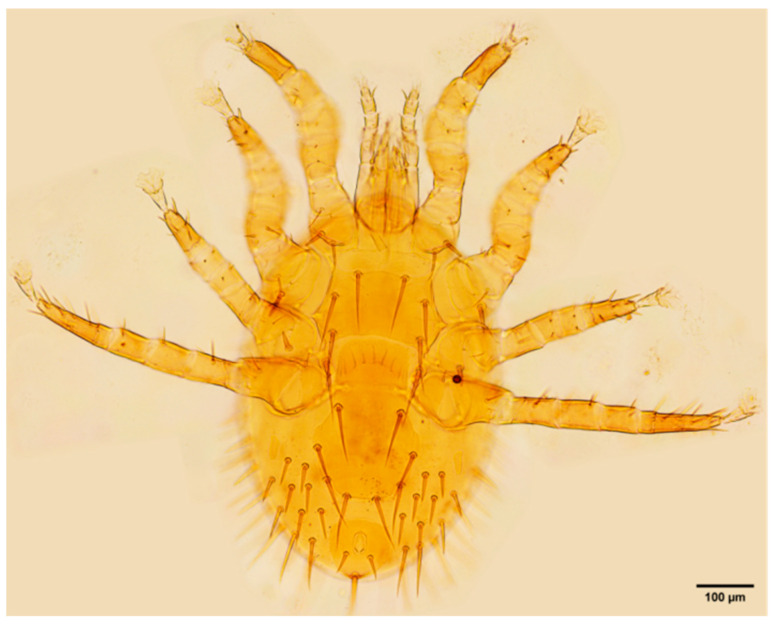
*Laelaps chini* photograph; female mite; center view (scale = 100 μm).

Of the 167 gamasid mite species identified, 22 species were the main ones, each with over 500 individuals. Six mite species were the most abundant, accounting for 76.59% (*C_r_* = 76.59%, 108,369/141,501) of all mites, making them the dominant species in the whole province (Table 5). These dominant species were *Laelaps nuttalli*, *L. echidninus*, *Dipolaelaps anourosorecis*, *L. guizhouensis*, *L. turkestanicus*, and *L. chini* (Figure A1, Figure A2, Figure A3, Figure A4, Figure A5 and Figure A6).

There were 13 vector species of gamasid mites found in Yunnan, which can serve as vectors or potential vectors of rickettsial pox, HFRS, and some other zoonoses (Table 6). The evidence for the 13 vector mite species preserving and transmitting the pathogens of some zoonotic diseases is based on previously published literature (Table 7) [55,56,57,58,59,60].

The majority of the identified gamasid mites were ectoparasitic species, being obligate or facultative ectoparasites (*C_r_* = 99.46%) [52,61,62,63,64]. A small number of gamasid mites were free-living species (*C_r_* = 0.54%) that might have accidentally crawled onto the body surfaces of small mammals (Table 8).

**Figure 5:**
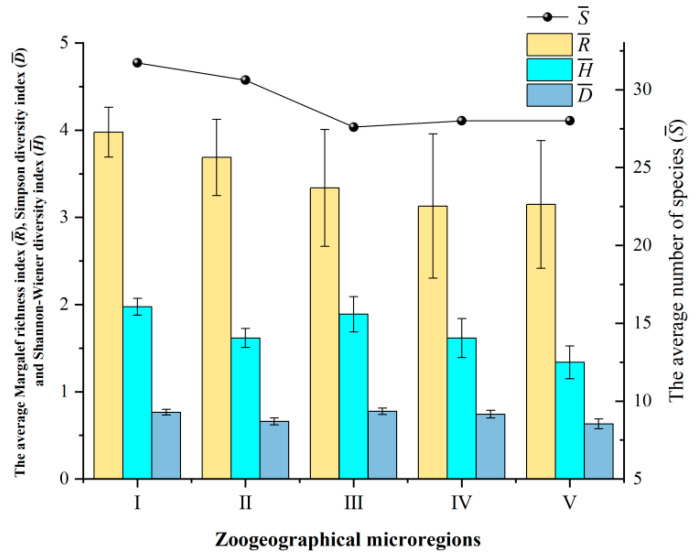
Average community indexes ($\bar{S}$, $\bar{R}$, $\bar{D}$, and $\bar{H}$) of gamasid mites in the five zoogeographical microregions of Yunnan Province, southwest China (1990–2022).

Among the five zoogeographical microregions, microregions I and II exhibited a higher average species diversity of gamasid mites compared to microregions III, IV, and V. Microregion I had the highest average number of species (31.73 species/500 hosts), an average Margalef richness index ($\bar{R}$ = 3.98), and an average Shannon–Wiener diversity index ($\bar{H}$ = 1.97). In contrast, microregion V showed the lowest $\bar{D}$ and $\bar{H}$ ($\bar{D}$ = 0.63, $\bar{H}$ = 1.34) (Table 9, Figure 5).

**Figure 6:**
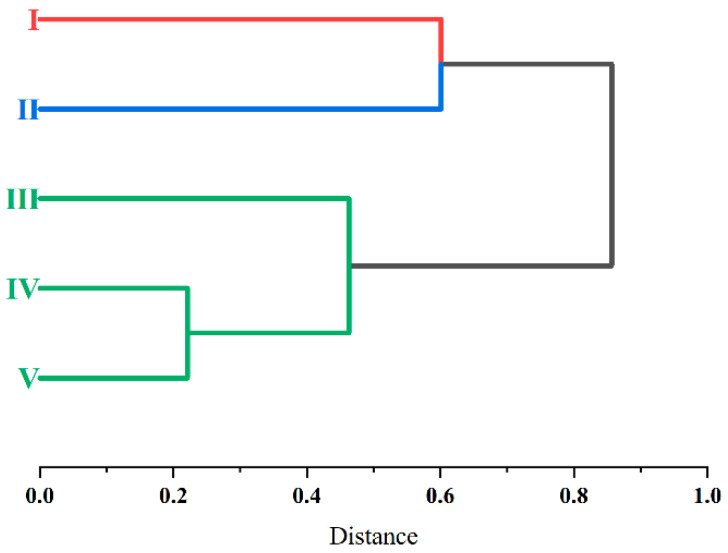
Similarity and hierarchical clustering dendrogram of gamasid mite faunae in different zoogeographical microregions of Yunnan Province, southwest China (1990–2022).

The dominant species composition of gamasid mites varied in the different zoogeographical microregions. For example, *D. anourosorecis* was the most abundant in microregion I, and *L. echidninus* was the most dominant mite species in microregion III (Table 10). A hierarchical clustering analysis was conducted to reveal the similarity among the faunae of gamasid mite communities on small mammal hosts in the five zoogeographical microregions, in which the average number of host species and gamasid mites, the overall infestation indexes of gamasid mites, the average community indexes ($\bar{S}$, $\bar{D}$, $\bar{H}$, $\bar{R}$) of hosts and gamasid mites, and the constituent ratios (*C_r_*) of dominant host species and gamasid mites were selected as variables. In the clustering dendrogram, the faunae of gamasid mite communities in microregions IV, V, and III had a high similarity and they clustered into one group, in which the communities of microregions IV and V showed the highest similarity. The mite faunae in microregions I and II also showed a high similarity and they clustered into one group (Figure 6).

### 3.3. Relationship Between Gamasid Mites and Their Hosts

**Figure 7:**
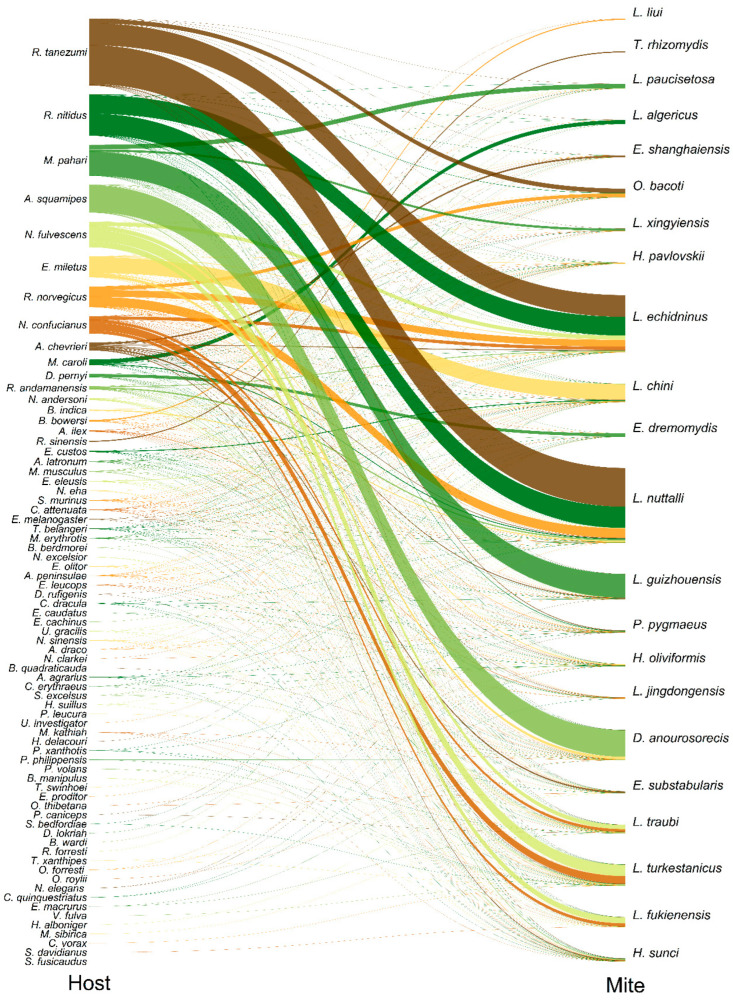
The mutual relationships between the 22 main gamasid mite species and their small mammal hosts in Yunnan Province of southwest China (1990–2022).

The parallel set diagram analysis revealed the intricate relationships between the 22 main gamasid mite species and their small mammal hosts, in which the color ribbon or color line represents the mutual relationships between different species of gamasid mites and their corresponding hosts, and the base width of the ribbons or lines represents the number of corresponding gamasid mites and hosts (Figure 7). The results reveal that a single host species can harbor several mite species, and a single mite species can parasitize several host species, indicating the low host specificity of gamasid mites.

### 3.4. Ecological Niches of Gamasid Mites

Based on the 40 survey sites (spatial resources), the spatial niches of 22 main gamasid mite species with 500 individuals were calculated. Six species of gamasid mites exhibited high spatial niche values (*B_i_* > 20.00), and *L*. *echidninus* had the highest niche value (*B_i_* = 28.95), which indicates the wide geographical distribution of the mites. *Tylolaelaps rhizomydis* exhibited the lowest spatial niche (*B_i_* = 2.50), revealing its narrow geographical distribution (Table 11).

**Figure 8:**
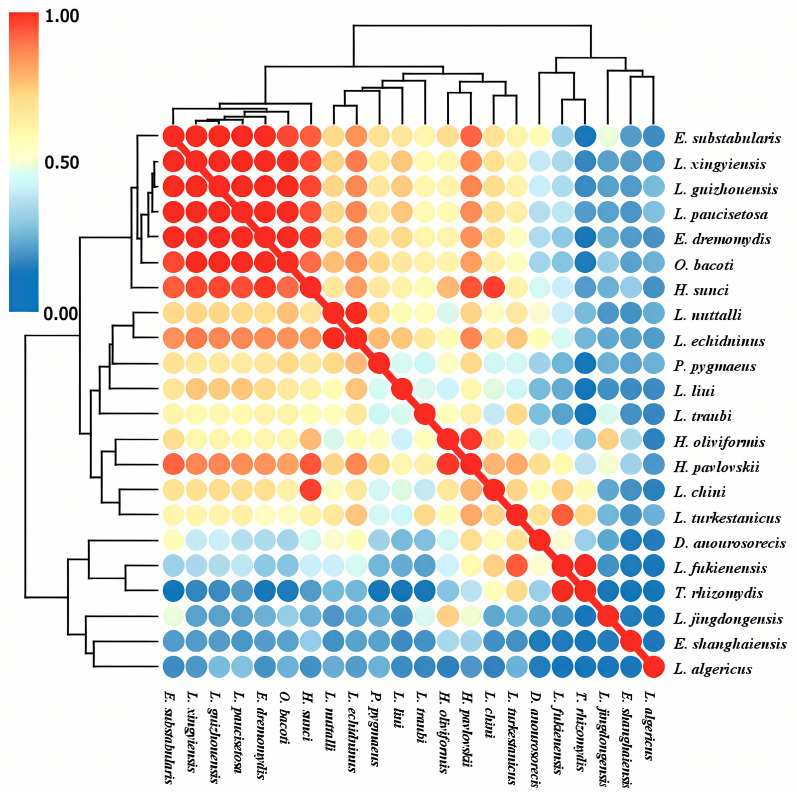
The dendrogram and heatmap visualization of ecological niche overlaps (host niche overlaps) of the 22 main gamasid mite species on 78 small mammal host species in Yunnan Province of southwest China (1990–2022).

When the 78 species of small mammal hosts were used as host resources, the host niches of 22 main gamasid mite species were calculated. Most gamasid mite species were collected from more than 10 host species (host range > 10), with relatively high host niche values (*B_i_* > 1.0), indicating they can select a wide range of hosts with low host specificity (Table 11). The dendrogram and heatmap in Figure 8 show the ecological niche overlaps (host niche overlaps) of 22 main gamasid mite species on 78 small mammal host species, in which different color circles represent the degrees of the niche overlaps. With overlap values ranging from 0 to 1, the color of the circles gradually changes from deep blue, light blue, yellow, pink, and light red to deep red. The deep red color indicates the highest niche overlap, and the deep blue color indicates the lowest or no niche overlap. Two species of gamasid mites, *Laelaps guizhouensis* and *L. xingyiensis*, exhibited the highest value of niche overlap, with *O_ik_* = 0.997. The overlap values were also high (*O_ik_*: 0.992–0.989) between every two *L. paucisetosa*, *L. guizhouensis*, *L. xingyiensis*, *Eulaelaps dremomydis*, *L. xingyiensis,* and *L. guizhouensis* species, and these gamasid mite species were clustered into an overlapped group (Figure 8).

**Figure 9:**
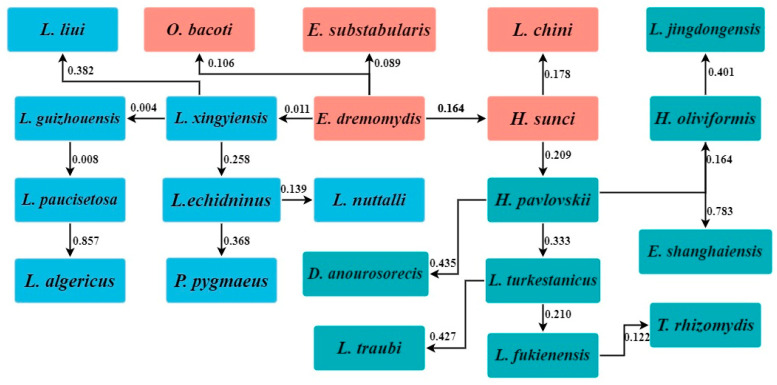
The minimum spanning tree (MST) of the spatial niche distances of 22 main gamasid mite species in 40 survey sites in Yunnan Province of southwest China (1990–2022).

The spatial niche overlap values of 22 main gamasid mite species in 40 survey sites were too different to be visualized using the dendrogram and heatmap. Instead, the minimum spanning tree (MST) was used to reveal the spatial niche distances of the gamasid mites, where corresponding values represent the niche distances of adjacent gamasid mite species. The smaller the value is, the shorter the spatial niche distance would be (indicating higher similarity), and vice versa. For example, *L. xingyiensis* and *L. guizhouensis* exhibited the lowest niche distance value (*d_ij_* = 0.004), indicating that these two mite species had the highest similarity in spatial niches. Similarly, *L. guizhouensis* and *L. paucisetosa* (*d_ij_* = 0.008), and *E. dremomydis* and *L. xingyiensis* (*d_ij_* = 0.011) also showed high similarity in spatial niches, indicating they had a similar geographical distribution (Figure 9).

### 3.5. Interspecific Relationships of Gamasid Mites

**Figure 10:**
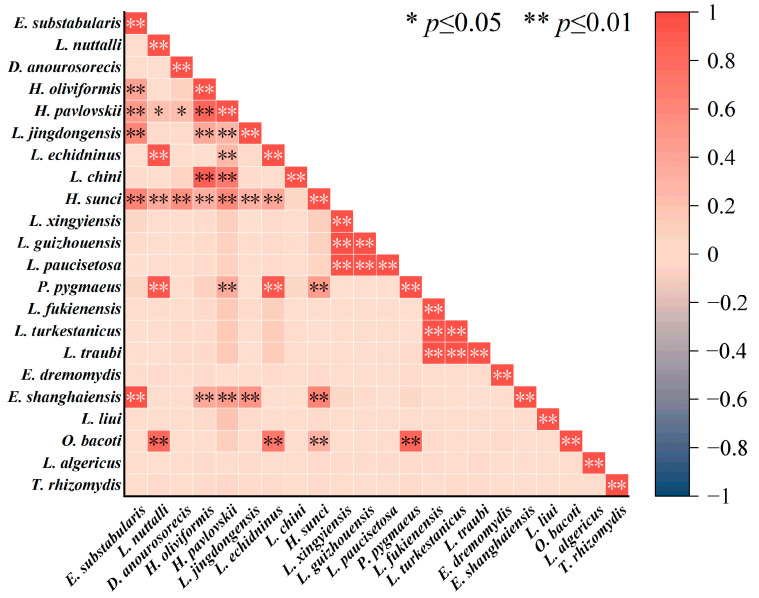
Interspecific relationships of 22 main gamasid mite species on small mammal hosts in Yunnan Province of southwest China (1990–2022).

The correlation coefficient (R) was used to measure the interspecific relationship between any two of the twenty-two main gamasid mite species and the seventy-eight species of small mammal hosts, and the result was visualized using a heatmap, with pink squares indicating positive correlations, and grayish blue ones squares indicating negative correlations (Figure 10). The depth of the color indicates the degree of positive or negative correlation. The values of correlation coefficient (R) for negative correlations ranged from −1 to 0 [−1, 0], and those for positive correlations ranged from 0 to 1 [0, 1]. In the selection of small mammal hosts, the 20 main species of gamasid mites were positively correlated with each other to varying degrees. For example, a positive correlation existed between *L. echidninus* and *L. nuttalli* (*R* = 0.97, *p* < 0.01), two dominant species of gamasid mites. *Laelaps echidninus* also exhibited a high positive correlation with *P. pygmaeus* (*R* = 0.96, *p* < 0.01). *Laelaps turkestanicus*, one of the dominant mite species, showed a high positive correlation with *L. traubi* (*R* = 0.99, *p* < 0.01) and *L. fukienensis* (*R* = 0.99, *p* < 0.01). *Laelaps guizhouensis*, another dominant mite species, also showed a high positive correlation with *L. xingyiensis* (*R* = 0.99, *p* < 0.01) and *L. paucisetosa* (*R* = 0.99, *p* < 0.01) (Figure 10).

### 3.6. Distribution of Gamasid Mites in Different Geographical Landscapes and Habitats

There are two distinct geographical landscape types (mountainous landscape and flatland landscape) and two distinct habitat types (indoor habitat and outdoor habitat) in Yunnan Province. In the present study, the indoor habitat includes human houses, poultry houses, pig pens, cattle pens, and stables, and the outdoor habitat includes farmland, scrubland, and woodland. A total of 18,063 small mammal hosts were captured across 40 survey sites in Yunnan Province. Of the 18,063 small mammal hosts, 12,827 had landscape records, and 5236 had no landscape records. Of the 12,827 hosts with landscape records, 8238 were captured in the mountainous landscape, and 4589 in the flatland landscape. The 5236 hosts without landscape records were excluded from the statistical analysis. Of the 18,036 small mammal hosts with habitat records, 14,335 hosts were captured in outdoor habitats, and 3701 in indoor habitats. The 27 hosts without habitat records were not included in the analysis. Due to the inconsistent numbers of host samples in different landscapes and habitats, every 500 hosts were grouped to calculate the average values of community indexes ($\bar{S}$, $\bar{D}$, $\bar{H}$, $\bar{R}$). The hosts with ≤250 <500 individuals constituted an independent last group. The hosts with <250 individuals merged into the former group.

**Figure 11:**
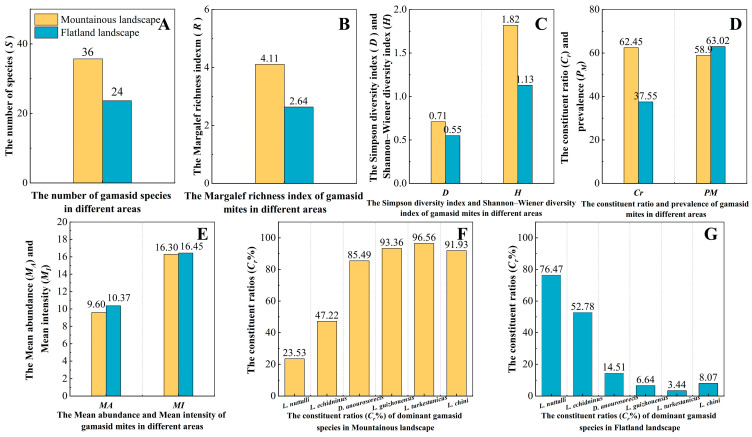
(**A**–**G**) The species composition, infestation indexes, and community diversity indexes of gamasid mites in different geographical landscapes of Yunnan Province.

**Figure 12:**
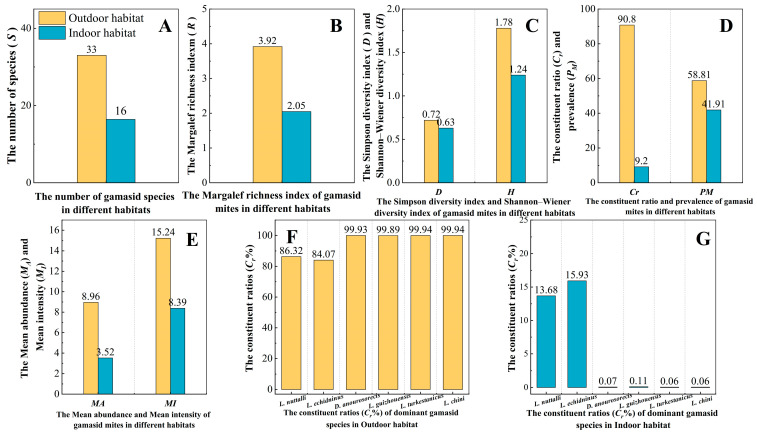
(**A**–**G**) The species composition, infestation indexes, and community diversity indexes of gamasid mites in different habitats of Yunnan Province.

The results showed that two average community diversity indexes of gamasid mites ($\bar{R}$, $\bar{H}$) per 500 hosts in the mountainous landscape and outdoor habitat were obviously higher than those in the flatland landscape and indoor habitat (landscape: $\bar{R}$: *p* = 0.024 < 0.05; $\bar{H}$: *p* = 0.002 < 0.01; habitat: $\bar{R}$: *p* = 0.002 < 0.01; $\bar{H}$: *p* = 0.005 < 0.01) (B and C in Figure 11 and Figure 12). The average number of gamasid mites ($\bar{S}$) in the outdoor habitat was higher than that in the indoor habitat (*p* = 0.004 < 0.01); the average number of gamasid mites ($\bar{S}$) in the mountainous landscape was also higher than that in the flatland landscape, but the difference was of no statistical significance (*p* = 0.054 > 0.05) (A in Figure 11 and Figure 12). The average Simpson diversity index ($\bar{D}$) in the mountainous landscape was higher than that in the flatland landscape (*p* = 0.017 < 0.05); the average Simpson diversity index ($\bar{D}$) in the outdoor habitat was also higher than that in the indoor habitat, but the difference was of no statistical significance (*p* = 0.065 > 0.05) (C in Figure 11 and Figure 12).

Although the constituent ratio (*C_r_*, %) in the mountainous landscape was obviously higher than that in the flatland landscape, the infestation indexes (*PM*, *MA*, *MI*) in the flatland landscape were slightly higher than those in the mountainous landscape (D, E in Figure 11). The composition and *C_r_* of dominant mite species in the flatland landscape were very different from those in the mountainous landscape (F, G in Figure 11). The constituent ratio (*C_r_*, %) and infestation indexes (*PM*, *MA*, *MI*) in the indoor habitat were obviously lower than those in the outdoor habitat (D, E in Figure 12). The composition and *C_r_* of the dominant mite species in the indoor habitat were very different from those in the outdoor habitat (F, G in Figure 12).

### 3.7. Estimation of Total Number of Gamasid Mite Species

Based on the Chao 1 estimation method, the expected total number of gamasid mite species in Yunnan Province was estimated to be 203 species (S* = 203.13), which is 36 species more than the 167 species identified in the actual survey.

## 4. Discussion

### 4.1. General Faunal Characteristics of Gamasid Mites in Yunnan of Southwest China

Gamasid mites are a large group of arthropods with a great many species. Many gamasid mites are free-living species that are widely distributed in various natural environments such as soil, humus, and garbage dump [32,65]. Some other gamasid mites are parasitic species, including endoparasitic and ectoparasitic ones [61,66]. Some gamasid mites belong to parasitic species, including endoparasitic and ectoparasitic ones, of which the ectoparasitic is the most common and important [63,64,67]. The present paper only involves the gamasid mites associated with rodents and other sympatric small mammals, most of which are ectoparasitic species. The results showed that 141,501 gamasid mites (not including 2197 unidentified ones) identified from small mammal hosts belong to 167 species and 41 genera in 14 families. Of the 167 mite species, *Laelaps nuttalli*, *L. echidninus*, *Dipolaelaps anourosorecis*, *L. guizhouensis*, *L. turkestanicus,* and *L. chini* were the six dominant species of gamasid mites in Yunnan, which accounted for 76.59% of all the mites. Of the six dominant mite species, *L. echidninus* can serve as the potential vector of some zoonotic diseases [68,69,70].

The results of the present study indicate that there are abundant species of gamasid mites with high species diversity in Yunnan Province of southwest China. The 167 species of gamasid mites identified from rodents and other sympatric small mammals (hosts) in Yunnan obviously exceed those recorded in other provincial regions of China, e.g., 102 species in Qinghai, 98 species in Shanxi, 78 species in Hubei, 69 species in Fujian, 62 species in Heilongjiang, 44 in Hebei, and 44 in Shandong [23,71,72,73,74,75]. The documented species in some other provincial regions even included some free-living ones collected from a variety of abiotic environments, not just from the body surfaces of small mammals [76,77,78]. The high species diversity of gamasid mites in Yunnan may be related to the following factors: (1) In this study, a total of 78 species of small mammal hosts (18,063 individuals) were captured from 40 survey sites in Yunnan. The high species diversity of gamasid mites in Yunnan Province is obviously related to the abundant number of species of small mammal hosts in the province. Known as China’s “Kingdom of Plants and Animals”, Yunnan is a mountainous region rich in animal sources, including rodents, other small mammals, and arthropods [10,11,12]. (2) Ectoparasitic gamasid mites and other ectoparasites (fleas, lice, ticks, chiggers, etc.) are poikilotherms, and their geographical distribution is affected by both host factors and abiotic environmental factors [79,80,81,82]. In addition to host factors, abiotic factors such as topography and climate also affect the distribution of ectoparasites [83,84,85]. The complex and diverse topography, climate types, and ecological environment in Yunnan are also important factors leading to the high species diversity of gamasid mites in this area. (3) The original data of this study came from long-term field surveys at 40 survey sites in Yunnan, and a total of 18,063 small mammal hosts were captured. A wide range of geographical surveys with abundant host samples can find more ectoparasites [84,86,87], which is one of the reasons for the high species diversity of gamasid mites in the present study. (4) In the present study, a small number of gamasid mites were free-living species (Table 8), which also increases the number of “ectoparasitic” gamasid mites to a certain extent.

Among the 167 gamasid mite species identified from Yunnan, 99.46% of them were obligate or facultative ectoparasites (*C_r_* = 99.46%), and only a small number of the mites were free-living species with *C_r_* = 0.54% (Table 8). These free-living species are mainly from the families Macronyssidae, Parasitidae, and Phytoseiidae [88,89,90]. Previous studies have shown that some free-living species of gamasid mites may accidentally “lose their way” and climb onto the body surface of other animals, and these are often collected from small mammals in actual field surveys [61,66,91,92]. Of the 18,063 small mammal hosts captured in Yunnan, the majority were rodents (the order Rodentia), accounting for 92.53% of all the hosts, and the four dominant host species (*R. tanezumi*, *A. chevrieri*, *E. miletus* and *R. norvegicus*) were all rodents (Table 2). This result is highly consistent with previous reports suggesting that rodents are the most common and important hosts of ectoparasitic gamasid mites [93,94,95,96]. The study of niches in this paper showed that most gamasid mite species were collected from more than 10 host species (host range > 10), with relatively high values of host niches (*B_i_* > 1.0) (Table 11). The complex relationships between gamasid mites and their hosts were intuitively visualized using the parallel set diagram analysis. The result showed that a single host species could harbor several mite species, and a single mite species could parasitize several host species (Figure 7). The above results indicate the low host-specificity of most gamasid mite species, which is also consistent with previous reports [61,93,97,98]. The low host-specificity of ectoparasitic gamasid mites means that a certain mite species could invade or bite different host species, rodents, and other small mammals. Through the biting activity of some vector mites with low host-specificity, some zoonotic diseases could be easily transmitted among different species of rodents and other wild animal hosts, and even from rodents to humans [99,100].

A total of 13 vector species of gamasid mites were found in Yunnan (Table 6), and these vector species can serve as vectors or potential vectors of rickettsial pox, HFRS, and some other zoonoses [5,6,55,56,57,58,59,60,94]. *L*. *echidninus* was one of the six dominant mite species in Yunnan, and the number of *L. echidninus* was the highest among the 13 vector species (*C_r_* = 59.79%, 27,482/45,963). In addition to invading humans to cause dermatitis, *L. echidninus* is suspected to be the potential vector of HFRS, rickettsial pox, Q fever, scrub typhus, leptospirosis, and some other zoonotic diseases (Table 7) [3,55,56,68,101,102]. Of the 13 vector mite species, *Ornithonyssus bacoti* accounted for 8.99% (*C_r_* = 8.99%, 4134/45,963) (Table 6), and it is of great medical importance. Apart from often causing dermatitis through biting humans, *O. bacoti* is an important vector or potential vector of rickettsial pox, HFRS, and some other zoonotic diseases (Table 7) [103,104,105,106]. In addition, the numbers of *Haemolaelaps glasgowi* (*C_r_* = 0.88%, 404/45,963) and *Tricholaelaps myonysognathus* (*C_r_* = 0.84%, 383/45,963) were also relatively high (Table 6), and they are also important potential vectors of HFRS and some other zoonotic diseases (Table 7) [68,106,107,108]. The occurrence of these vector mite species in Yunnan, combined with the low host-specificity of the mite, may increase the potential risk of zoonosis transmission and its persistence within this region [109,110,111].

### 4.2. Faunal Similarity of Gamasid Mites in Different Zoogeographical Microregions

In terms of its zoogeography, Yunnan belongs to the Sino–Indian Subrealm in the Oriental Realm with two regions, two subregions, and five microregions: the Middle Microregion of the Hengduan Mountains (I), the Southern Microregion of the Hengduan Mountains (II), the Eastern Plateau Microregion of Yunnan (III), the Western Plateau Microregion of Yunnan (IV), and the Southern Mountainous Microregion of Yunnan (V) [12,36,111]. In the clustering dendrogram of the present study, the faunae of gamasid mite communities in microregions IV, V, and III had high similarity and they clustered into one group, in which the communities of microregions IV and V showed the highest similarity. The mite faunae in microregions I and II also showed a high similarity and they clustered into one group (Figure 6). The microregions I and II exhibited high species diversity of gamasid mites with high average community indexes than other three microregions (III, IV, and V) (Table 9). The dominant species composition of gamasid mites varied in different zoogeographical microregions. *Dipolaelaps anourosorecis* was the most abundant in microregion I, and *L. echidninus* was the most dominant in microregion III (Table 10). Yunnan Province in southwest China is a mountainous region with a vast territory, in which the average altitudes gradually decrease from northwest to southeast. The northwestern part of Yunnan has the highest average altitude, complex topography and ecological environment, and diverse climate types. In contrast, the average altitude of southern Yunnan is obviously lower, and the topography, climate type and ecological environment are much simpler than those of northwestern Yunnan [12,112,113]. Located in southern Yunnan, microregions IV and V are adjacent areas with a much lower altitude, simpler topography, and warmer climate than microregions I and II in northwestern Yunnan [114]. In terms of zoogeography, the microregions IV and V belong to the same subregion, the “Southern Yunnan Mountain Subregion” of the “South China Region” (Table 1) [111,115]. The high similarity in the gamasid mite faunae between microregions IV and V may be related to their adjacent geographical location, and similar topography and climate types. Microregions I and II are adjacent to each other in the Hengduan Mountainous region [36,66] and belong to the same subregion, the “Southwest Mountain Subregion” of the “Southwest China Region” (Table 1) [111,115], which may be related to the similarity between the gamasid fauna in these two microregions. Located in the east of Yunnan, microregion III belongs to the Yunnan–Guizhou Plateau [66,116], and the fauna of gamasid mites in the microregion III showed a high dissimilarity with the faunae in microregions I and II (Figure 1 and Figure 6). Of the five microregions, microregion I has the most complicated topography, and the most diverse ecological environment and climate types. Most of microregion I is located within a very unique geographical region, the Three Parallel Rivers Area, where three rivers (Jinshajiang, Lancangjiang and Nujiang rivers) flow from the north to the south without intersection. The highly diverse topography, landscape, ecological environments, and climate types in the Three Parallel Rivers Area lead to high species diversity in the plants and animals in this area [14,66,117]. The specific geographical location of microregion I could be why it has the highest species diversity of gamasid mites of all the microregions (Table 9).

### 4.3. Distribution of Gamasid Mites in Different Geographical Landscapes and Habitats

Although Yunnan Province is generally a mountainous region, there are flatlands dotted around the vast territory of the province. The present study used the average community indexes ($\bar{S}$, $\bar{D}$, $\bar{H}$, $\bar{R}$) to compare the community diversity of two geographical landscapes and habitats. The results showed that two community diversity indexes ($\bar{H}$, $\bar{R}$) in the mountainous landscape and outdoor habitat were obviously higher than those in the flatland landscape and indoor habitat (*p* < 0.05) (Figure 11 and Figure 12). In addition, the infestation indexes (*PM*, *MA*, *MI*) and the composition of dominant mite species in the mountainous landscape and outdoor habitat were also different from those in the flatland landscape and indoor habitat (Figure 11 and Figure 12). The results reveal the heterogeneity of the gamasid mite community in different environments, which suggests that, in addition to the host factor, the landscape and habitat also indirectly influence the distribution of gamasid mites. In comparison with the simplified environments in the flatland landscape and indoor habitat, the complex environments in the mountainous landscape and outdoor habitat would lead to a high community diversity among ectoparasites (including gamasid mites) and their hosts [118,119]. The wide variety of host species in the mountainous landscape and outdoor habitat provide diverse ecological niches for gamasid mites and other ectoparasites [120]. In contrast, the simplified environmental conditions and limited host availability are obviously related to the low community diversity of gamasid mites in the flatland landscape and indoor habitat [121]. The results of the present study are consistent with some previous reports on the arthropod communities associated with small mammals [66]. For instance, Krasnov reported that flea diversity among small mammals was high in regions with widespread habitat heterogeneity [122]. In comparison with indoor habitats, outdoor habitats supported the high species richness and diversity of mite communities found in other regions of China [3,91].

### 4.4. Estimation of Total Species of Gamasid Mites

The Chao 1 method used in the present study is a simple and commonly used way to roughly estimate the total number of species in a certain community or within a specific geographical region [53,54]. Based on Chao 1, the expected total number of gamasid mite species in Yunnan Province was estimated to be 203 species (S* = 203.13), which is 36 species more than the 167 species identified in the actual survey. This indicates that some rare species of gamasid mites were missed in the actual field survey. It is believed that some rare species are so few in number that they are very difficult to find in a certain collection [4,123]. In a specific field investigation, it is impossible to expand the collection indefinitely and collect all species. Therefore, it is necessary to scientifically estimate the expected total number of species in a specific geographical region [124,125].

## 5. Conclusions

In Yunnan Province of southwest China, which belongs to the Sino–Indian Subrealm in the Oriental Realm with two regions, two subregions, and five microregions, there are a large number of gamasid mites on the body surface of rodents and other sympatric small mammals and the mite species diversity is very high. The hosts of gamasid mite cross different orders, families, genera and species of small mammals, and rodents are the most important hosts. Most mite species have low host specificity, and they can simultaneously parasitize more than 10 host species. There are six common and dominant mite species in Yunnan, including *Laelaps nuttalli*, *L. echidninus*, *Dipolaelaps anourosorecis*, *L. guizhouensis*, *L. turkestanicus,* and *L. chini*. The species composition of gamasid mites exhibit environmental heterogeneity with higher species diversity in mountainous and outdoor habitats than in flatland/indoor environments. The co-existence of multiple vector mite species with low host specificity increases the potential risk of zoonosis transmission and focus persistence. The high niche overlap and positive interspecific correlation among some mite species indicates co-occurrence trends on shared hosts. In biogeographic patterns, the western/southern plateaus (microregions IV and V) have high similarity of mite community, and the Hengduan Mountains (I) exhibits unique mite diversity. Chao 1 estimation is a simple way to roughly estimate the expected total mite species in a certain geographical region. The key findings highlight the interplay of biogeography, host ecology, and environmental factors in shaping mite distributions, with implications for zoonotic disease surveillance in biodiverse regions.

## Figures and Tables

**Table 1:** The zoogeographical divisions of Yunnan Province in southwest China.

Realm and Subrealm	Regions	Subregions	Microregions	Names of Survey Sites
Sino-Indian Subrealm in Oriental Realm	Southwest China Region	Southwest Mountain Subregion	I—Middle Microregion of Hengduan Mountains	Gongshan, Jianchuan, Yulong, Xianggelila Lanping, Weixi, Deqin, Lushui, Fugong, Heqing, Yangbi, Longyang, and Tengchong
			II—Southern Microregion of Hengduan Mountains	Gengma, Yongde, Ninger, Yuanjiang, Dali, Weishan, and Nanjian
			III—Eastern Plateau Microregion of Yunnan	Luliang, Fuyuan, Suijiang, Wenshan, Qiaojia, Mengzi, Binchuan, Xiangyun, and Qiubei
	South China Region	Southern Yunnan Mountain Subregion	IV—Western Plateau Microregion of Yunnan	Longchuan, Lianghe, Ruili, and Yingjiang
			V—Southern Mountainous Microregion of Yunnan	Hekou, Simao, Maguan, Menghai, Jinghong, Jinping, and Cangyuan

**Table 2:** The list of 78 species of small mammal hosts identified from 40 survey sites (counties) of Yunnan Province, southwest China, between 1990 and 2022.

Taxonomic Taxa of Small Mammal Hosts	Collected Species and Individuals of Small Mammal Hosts (the Figures in the Brackets Are the Collected Individuals for Each Host Species).
**Rodentia**	Total hosts: 16,714 individuals; 50 species; 22 genera; 5 families
Muridae	*Apodemus chevrieri* (2216); *A. ilex* (566); *A. latronum* (341); *A. peninsulae* (94); *A. draco* (2); *A. agrarius* (4); *Bandicota indica* (28); *Berylmys bowersi* (57); *B. berdmorei* (3); *B. manipulus* (1); *Hapalomys delacouri* (1); *Leopoldamys edwardsi* (1); *Micromys erythrotis* (81); *Mus pahari* (739); *M. caroli* (489); *M. musculus* (223); *Niviventer confucianus* (582); *N. fulvescens* (288); *N. andersoni* (65); *N. eha* (36); *N. excelsior* (6); *Rattus tanezumi* (5387); *R. norvegicus* (1452); *R. nitidus* (998); *R. andamanensis* (348); *Vernaya fulva* (1)
Cricetidae	*Neodon clarkei* (15); *Eothenomys miletus* (2175); *E. custos* (105); *E. eleusis* (79); *E. proditor* (23); *E. cachinus* (19); *E. melanogaster* (12); *E. olitor* (7)
Pteromyidae	*Hylopetes alboniger* (6); *Petaurista caniceps* (5); *P. xanthotis* (5); *P. philippensis* (4); *Pteromys volans* (11); *Trogopterus xanthipes* (18)
Sciuridae	*Callosciurus erythraeus* (32); *C. quinquestriatus* (6); *Dremomys pernyi* (132); *D. lokriah* (5); *D. rufigenis* (3); *Rupestes forresti* (19); *Sciurotamias davidianus* (4); *Tamiops swinhoei* (18)
Spalacidae	*Rhizomys pruinosus* (1); *R. sinensis* (1)
**Eulipotyphla**	Total hosts: 1084 individuals; 21 species; 13 genera; 3 families
Talpidae	*Parascaptor leucura* (4); *Scaptonyx fusicaudus* (3); *Uropsilus gracilis* (32); *U. investigator* (17)
Erinaceidae	*Neotetracus sinensis* (12); *Hylomys suillus* (6)
Soricidae	*Anourosorex squamipes* (375); *Blarinella wardi* (9); *B. quadraticauda* (3); *Crocidura attenuata* (205); *C. dracula* (18); *C. vorax* (1); *Chimarrogale styani* (1); *C. himalayica* (1); *Episoriculus caudatus* (51); *E. leucops* (23); *E. macrurus* (11); *Nectogale elegans* (4); *Suncus murinus* (252); *Sorex excelsus* (36); *S. bedfordiae* (20);
**Carnivora**	Total hosts: 5 individuals; 2 species; 1 genus; 1 family
Mustelidea	*Mustela kathiah* (4); *M. sibirica* (1)
**Lagomorpha**	Total hosts: 38 individuals; 4 species; 1 genus; 1 family
Ochotonidae	*Ochotona forresti* (18); *O. thibetana* (11); *O. gaoligongensis* (8); *O. roylii* (1)
**Scandentia**	Total hosts: 222 individuals; 1 species; 1 genus; 1 family
Tupaiidae	*Tupaia belangeri* (222)

**Table 3:** Average community indexes of small mammal hosts in the five zoogeographical microregions of Yunnan Province, southwest China (1990–2022).

Zoogeographical Microregions	Species and Individuals of Small Mammal Hosts		Average Community Indexes of Small Mammal Hosts			
	No. of Species	Individuals	$\bar{S}$	$\bar{R}$	$\bar{D}$	$\bar{H}$
I	65	5541	15.09	2.26	0.70	1.68
II	43	6345	10.31	1.51	0.50	1.14
III	25	2327	12.60	1.88	0.73	1.67
IV	23	1394	13.00	1.97	0.57	1.30
V	24	2456	11.20	1.65	0.34	0.84
Total	78	18,063	-	-	-	-

Annotation: I = Middle Microregion of the Hengduan Mountains; II = Southern Microregion of the Hengduan Mountains; III = Eastern Plateau Microregion of Yunnan; IV = Western Plateau Microregion of Yunnan; V = Southern Mountainous Microregion of Yunnan. The average community indexes ($\bar{S}$, $\bar{R}$, $\bar{D}$, and $\bar{H}$) are the average values per 500 individual hosts. $\bar{S}$ = average number of species; $\bar{R}$ = average Margalef richness index; $\bar{D}$ = average Simpson diversity index; $\bar{H}$ = average Shannon–Wiener diversity index.

**Table 4:** The dominant species of small mammal hosts in five zoogeographical microregions in Yunnan Province, southwest China (1990–2022).

Microregions	Names of Dominant Hosts	Numbers and Constituent Ratios (*C_r_*) of Hosts		Microregions	Names of Dominant Hosts	Numbers and Constituent Ratios (*C_r_*) of Hosts	
		No.	*C_r_*, %			No.	*C_r_*, %
I	*A. chevrieri*	1206	21.77	IV	*R. tanezumi*	833	59.76
	*E. miletus*	747	13.48		*E. miletus*	162	11.62
	*R. tanezumi*	622	11.23	V	*R. tanezumi*	1848	75.24
	*A. ilex*	566	10.21				
II	*R. tanezumi*	1593	25.11	Total	*R. tanezumi*	5387	29.82
	*E. miletus*	1209	19.05	(I–V)	*A. chevrieri*	2216	12.27
	*A. chevrieri*	782	12.32		*E. miletus*	2175	12.04
III	*R. norvegicus*	670	28.79		*R. norvegicus*	1452	8.04
	*R. tanezumi*	491	21.1				
	*Mus caroli*	248	10.66				

**Table 5:** The list of 167 species of gamasid mites identified from small mammal hosts in Yunnan Province, southwest China, between 1990 and 2022.

Taxonomic Taxa of Gamasid Mites	Collected Species and Individuals of Gamasid Mites (the Figures in the Brackets Are the Collected Individuals for Each Mite Species).
**Laelapidae**	135,110 individuals; 107 species; 17 genera.
*Laelaps*	*L. nuttalli* (35,683); *L. echidninus* (27,482); *L. guizhouensis* (11,985); *L. turkestanicus* (10,196); *L. traubi* (4025); *L. chini* (8706); *L. paucisetosa* (2231); *L. algericus* (1998); *L. xingyiensis* (1190); *L. fukienensis* (4679); *L. liui* (533); *L. jettmari* (425); *L. hongaiensis* (2); *L. jingdongensis* (622); *L. extremi* (14); *L. clethrionomydis* (17); *L. taingueni* (2); *L. hilaris* (96); *L. micromydis* (7); *L. sedlaceki* (1)
*Haemolaelaps*	*H. glasgowi* (404); *H. casalis* (50); *H. petauristae* (5); *H. anomalis* (1); *H. chinensis* (11); *H. traubi* (83); *H. cordatus* (12); *H. semidesertus* (2); *H. orientalis* (27); *H. liae* (2); *H. triangular* (6)
*Androlaelaps*	*A. singularis* (177); *A. hsui* (29); *A. subpavlovskii* (6); *A. singuloides* (7)
*Dipolaelaps*	*D. anourosorecis* (14,317); *D. jiangkouensis* (98); *D. longisetosus* (4); *D. chimmarogalis* (7); *Dipolaelaps hoi* (5)
*Hyperlaelaps*	*H. microti* (72)
*Neocypholaelaps*	*N. indica* (2)
*Tricholaelaps*	*T. myonysognathus* (384)
*Hypoaspis*	*H. pavlovskii* (545); *H. miles* (143); *H. lubrica* (166); *H. chianensis* (61); *H. praesternalis* (20); *H. concinna* (356); *H. aculeifer* (2); *H. ovatus* (238); *H. digitalis* (5); *H. tengi* (9); *H. chelaris* (1); *H. hrdyi* (13); *H. haiyuanensis* (1); *H. qinghaiensis* (5); *H. lecae* (16); *H. sorecis* (12); *H. vacua* (2)
*Ololaelaps*	*O. Veneta* (1)
*Haemogamasus*	*H. oliviformis* (914); *H. dorsalis* (271); *H. gongshanensis* (259); *H. monticola* (150); *H. sexsetosus* (123); *H. pontiger* (40); *H. multidentis* (69); *H. quadrisetatus* (17); *H. yunlongensis* (3); *H. paradauricus* (36); *H. sanxiaensis* (110); *H. hodosi* (4); *H. trifurcisetus* (41); *H. quadratus* (4); *H. szechwanensis* (19); *H. nidiformis* (8); *H. dauricus* (2); *H. gui* (6); *H. nidi* (4); *H. emeiensis* (20); *H. parascaptoris* (11)
*Eulaelaps*	*E. stabularis* (43); *E. shanghaiensis* (815); *E. dremomydis* (1524); *E. substabularis* (911); *E. huzhuensis* (164); *E. silvestris* (6); *E. dongfangis* (3); *E. tsinghaiensis* (5); *E. novus* (12)
*Gymnolaelaps*	*G. sinensis* (4); *G. weishanensis* (46)
*Cosmolaelaps*	*C. retirugi* (34); *C. yeruiyuae* (71)
*Hirstionyssus*	*H. sunci* (1497); *H. callosciuri* (31); *H. neosinicus* (8); *H. qinghaiensis* (1); *H. microti* (4); *H. isabellinus* (11); *H. musculi* (64); *H. hupehensis* (1); *H. phodopi* (2)
*Mysolaelaps*	*M. cunicularis* (1)
*Qinghailaelaps*	*Q. gui* (7)
*Tylolaelaps*	*T. rhizomydis* (523)
**Dermanyssidae**	388 individuals; 2 species; 2 genera
*Liponyssoides*	*L. muris* (295)
*Echinonyssus*	*E. nasatus* (93)
**Macronyssidae**	4135 individuals; 2 species; 2 genera
*Ornithonyssus*	*O. bacoti* (4134)
*Macronyssus*	*M. hongheensis* (1)
**Aceosejidae**	207 individuals; 14 species; 1 genus
*Lasioseius*	*L. medius* (97); *L. trifurcipilus* (19); *L. qinghaiensis* (38); *L. multispathus* (1); *L. liaohaorongae* (8); *L. chenpengi* (1); *L. analis* (12); *L. praevius* (17); *L. jilinensis* (6); *L. paucispathus* (1); *L. confusus* (4); *Lasioseius spatulus* (1); *L. sugawari* (1); *L. daanensis* (1)
**Ameroseiidae**	8 individuals; 3 species; 2 genera
*Ameroseius*	*A. taoerhensis* (3); *A. denticulatus* (3)
*Sinoseius*	*S. lobatus* (2)
**Parasitidae**	90 individuals; 12 species; 3 genera
*Parasitus*	*P. consanguineus* (33); *P. wangdunqingi* (8); *P. baoshanensis* (1); *P. wentinghuani* (5); *P. fragilis* (1); *P. quadrichaetus* (2)
*Vulgarogamasus*	*V. xiphoideus* (2); *V. oudemansi* (1); *V. xinjiangensis* (4); *V. radialis* (1); *V. plumosus* (31)
*Amblygamasus*	*A. shennongjiaensis* (1)
**Phytoseiidae**	1 individual; 1 species; 1 genus
*Amblyseius*	*A. ishizuchiensis* (1)
**Ascidae**	6 individuals; 3 species; 3 genera
*Asca*	*A. idiobasis* (4)
*Antennoseius*	*A. sinicus* (1)
*Paraproctolaelaps*	*P. zhongweiensis* (1)
**Parholaspidae**	6 individuals; 4 species; 2 genera
*Parholaspis*	*P. wuhanensis* (1)
*Gamasholaspis*	*G. eothenomydis* (2); *G. concavus* (2); *G. imiteothenomydis* (1)
**Macrochelidae**	447 individuals; 10 species; 3 genera
*Macrocheles*	*M. liguizhenae* (389); *M. muscaedomesticae* (37); *M. sinicus* (4); *M. plateculus* (1); *M. glaber* (8); *M. plumiventris* (2); *M. merdarius* (1); *M. nataliae* (3)
*Glyptholaspis*	*G. wuhouyongi* (1)
*Holostaspella*	*H. ornata* (1)
**Pachylaelaptidae**	17 individuals; 3 species; 1 genus
*Pachylaelaps*	*P. badongensis* (1); *P. nuditectus* (14); *P. xizangensis* (2)
**Rhodacaridae**	2 individuals; 2 species; 2 genera
*Euryparasitus*	*E. laxiventralis* (1)
*Rhodacarellus*	*R*. *liuzhiyingi* (1)
**Eviphididae**	1 individual; 1 species; 1 genus
*Eviphis*	*E. wanglangensis* (1)
**Blattisocidae**	1093 individuals; 3 species; 1 genus
*Proctolaelaps*	*P. pygmaeus* (1079); *P. yinchuanensis* (1); *P. pistilli* (13)

**Table 6:** The statistics for 13 vector species of gamasid mites in Yunnan Province of southwest China (1990–2022).

Names of Vector Gamasid Mite Species	Numbers and Constituent Ratios (*C_r_*,) of the Mites		Number of Host Species (Host Range)	Distribution in Different Microregions
	No.	*C_r_*%		
*Laelaps* *echidninus*	27,482	59.79	25	I, II, III, IV, V
*L.* *turkestanicus*	10,196	22.18	35	I, II, III, IV, V
*L. algericus*	1998	4.35	7	I, II, III, IV, V
*L. jettmari*	425	0.92	10	I, II, III
*L. clethrionomydis*	17	0.04	5	I, II, III
*Haemolaelaps glasgowi*	404	0.88	14	I, II, III, V
*H. casalis*	50	0.11	13	I, II, III, IV, V
*Tricholaelaps myonysognathus*	384	0.84	12	I, II, III, IV, V
*Haemogamasus nidi*	4	0.01	3	I, II, V
*Eulaelaps stabularis*	43	0.09	10	I, II, III, V
*E. shanghaiensis*	815	1.77	11	I, II, III
*Hirstionyssus isabellinus*	11	0.02	5	I, II, III, V
*Ornithonyssus bacoti*	4134	8.99	15	I, II, III, IV, V
Total	45,963	100.00	-	-

**Table 7:** The evidence for the 13 vector gamasid mite species preserving and transmitting the pathogens of some zoonotic diseases according to the previously published literature [55,56,57,58,59,60].

Names of Vector Gamasid Mites	Rickettsial Pox	HFRS	TBE	LCM	Psittacosis	Endemic Typhus	Q Fever	North-Asian Tick-Borne Typhus	Scrub Typhus	Tularemia	Plague	Leptospirosis
*L.* *echidninus*	#	▲					#		#			#
*L.* *turkestanicus*									#			
*L. algericus*				#							#	
*L. jettmari*												
*L. clethrionomydis*			#							#		
*H. glasgowi*		#▲◯	#	#			▲	#		#▲		
*H. casalis*					#		#	#				
*T. myonysognathus*		#▲					#					
*H. nidi*		#	#	#▲			▲			#		
*E. stabularis*		#▲◯		#								
*E. shanghaiensis*												
*H. isabellinus*		#	▲	#				#		◯		
*O. bacoti*	#◯	#▲		▲		#◯	◯			◯		▲

Annotations: # the pathogens of corresponding zoonotic diseases were detected in the gamasid mites; ▲ the gamasid mites can preserve and transmit the pathogens through laboratory experiments; ◯ besides preserving and transmitting the pathogens, the gamasid mites can cause transovarial transmission in the laboratory. Abbreviations: HFRS = hemorrhagic fever with renal syndrome; TBE = tick-borne encephalitis; LCM = lymphocytic choriomeningitis.

**Table 8:** Ectoparasitic and free-living gamasid mites identified from rodents and other sympatric small mammals in Yunnan Province, southwest China (1990–2022).

Different Groups of Gamasid Mites	No. of Species and Constituent Ratio (*C_r_*, %) of Gamasid Mites		No. of Individuals and Constituent Ratio (*C_r_*, %) of Gamasid Mites		Corresponding Families of Gamasid Mites in Zoological Taxonomy
	No.	*C_r_*, %	No.	*C_r_*, %	
Ectoparasitic group	129	77.25	140,738	99.46	Laelapidae, Dermanyssidae, Macronyssidae, Ameroseiidae, Ascidae, Parholaspidae, Rhodacaridae, Blattisocidae
Free-living group	38	22.75	763	0.54	Macrochelidae, Parasitidae, Phytoseiidae, Aceosejidae, Pachylaelaptidae, Eviphididae
Total	167	100	141,501	100	14 families

**Table 9:** Average community indexes of gamasid mites in the five zoogeographical microregions of Yunnan Province, southwest China (1990–2022).

Zoogeographical Microregions	The Numbers of Species and Individuals of Gamasid Mites		The Average Number of Species $\left(\bar{S}\right)$, Average Margalef Richness Index $\left(\bar{R}\right)$, Average Simpson Diversity Index $\left(\bar{D}\right)$, and Average Shannon–Wiener Diversity Index ($\bar{H}$)			
	No. of Species	Individuals	$\bar{S}$	$\bar{R}$	$\bar{D}$	$\bar{H}$
I	108	34,932	31.73	3.98	0.77	1.97
II	105	50,413	30.62	3.69	0.66	1.62
III	72	14,523	27.60	3.34	0.78	1.89
IV	49	15,760	28.00	3.13	0.74	1.62
V	77	25,873	28.00	3.15	0.63	1.34
Total	167	141,501	-	-	-	-

Annotation: I, II, III, IV, V, $\bar{S}$, $\bar{R}$, $\bar{D}$ and $\bar{H}$, same as in Table 2.

**Table 10:** The dominant species of gamasid mites in five zoogeographical microregions in Yunnan Province, southwest China (1990–2022).

Microregions	Names of Dominant Gamasid Mites	Numbers and Constituent Ratios (*C_r_*)% of Gamasid Mites		Microregions	Names of Dominant Gamasid Mites	Numbers and Constituent Ratios (*C_r_*)% of Gamasid Mites	
		No.	*C_r_*, %			No.	*C_r_*, %
I	*D.* *anourosorecis*	8622	24.68	IV	*L.* *nuttalli*	3341	21.20
	*L.* *nuttalli*	7410	21.21		*D.* *anourosorecis*	2906	18.44
	*L.* *echidninus*	5115	14.64		*L.* *echidninus*	2318	14.71
II	*L.* *nuttalli*	11,092	22.00	V	*L.* *nuttalli*	11,357	43.90
	*L.* *echidninus*	8767	17.39		*L.* *echidninus*	8066	31.18
	*L.* *guizhouensis*	8615	17.09	Total (I–V)	*L.* *nuttalli*	35,683	25.22
III	*L.* *echidninus*	3216	22.14		*L.* *echidninus*	27,482	19.42
	*L.* *nuttalli*	2483	17.10		*D.* *anourosorecis*	14,317	10.12
	*L.* *turkestanicus*	1916	13.19		*L.* *guizhouensis*	11,985	8.47

**Table 11:** Spatial and host niches of 22 main gamasid mite species in Yunnan Province of southwest China (1990–2022).

Names of Gamasid Mites	Individuals of Gamasid Mites	Constituent Ratio (*C_r_*) % of Gamasid Mites	Spatial Niches (*B_i_*) of Gamasid Mites		Host Niches (*B_i_*) and Host Ranges of Gamasid Mites	
			*B_i_*	Numbers of Collected Sites	*B_i_*	Host Ranges
*L.* nuttalli *	35,683	25.22	27.08	37	3.49	32
*L.* echidninus *	27,482	19.42	28.95	37	4.73	25
*D.* anourosorecis *	14,317	10.12	6.46	14	1.60	29
*L.* guizhouensis *	11,985	8.47	4.87	18	1.44	21
*L.* turkestanicus *	10,196	7.21	22.95	22	3.13	35
*L.* chini *	8706	6.15	12.32	24	1.74	34
*L. fukienensis*	4679	3.31	10.74	13	2.83	19
*O. bacoti*	4134	2.92	11.77	27	2.98	15
*L. traubi*	4025	2.84	12.68	16	3.50	27
*L. paucisetosa*	2231	1.58	5.36	14	1.41	11
*L. algericus*	1998	1.41	4.27	13	1.45	7
*E. dremomydis*	1524	1.08	3.03	8	1.42	14
*H. sunci*	1497	1.06	12.96	30	8.41	31
*L.* *xingyiensis*	1190	0.84	4.58	17	1.64	11
*P. pygmaeus*	1079	0.76	26.07	29	6.18	25
*H. oliviformis*	914	0.65	21.52	19	5.05	29
*E. substabularis*	911	0.64	7.88	13	2.24	21
*E. shanghaiensis*	815	0.58	6.57	11	1.52	11
*L. jingdongensis*	622	0.44	7.95	5	4.59	12
*H. pavlovskii*	545	0.39	23.04	34	11.57	26
*L. liui*	533	0.38	12.32	10	1.34	3
*T. rhizomydis*	523	0.37	2.50	1	1.28	1

Annotation: The species marked with “*” are the most abundant species with high constituent ratios (*C_r_*).

## Data Availability

The data presented in this study are available on request from the corresponding author due to intellectual property status.
